# Transcriptome analysis of a thermophilic and hydrogenogenic carboxydotroph *Carboxydothermus pertinax*

**DOI:** 10.1007/s00792-019-01091-x

**Published:** 2019-04-03

**Authors:** Yuto Fukuyama, Kimiho Omae, Takashi Yoshida, Yoshihiko Sako

**Affiliations:** Division of Applied Biosciences, Graduate School of Agriculture, Kyoto University, Kyoto, 606-8502 Japan

**Keywords:** Hydrogenogenic carboxydotroph, Carbon monoxide dehydrogenase, RNA sequencing, *Carboxydothermus pertinax*

## Abstract

A thermophilic and hydrogenogenic carboxydotroph, *Carboxydothermus pertinax,* performs hydrogenogenic CO metabolism in which CODH-II couples with distally encoded ECH. To enhance our knowledge of its hydrogenogenic CO metabolism, we performed whole transcriptome analysis of *C. pertinax* grown under 100% CO or 100% N_2_ using RNA sequencing. Of the 2577 genes, 36 and 64 genes were differentially expressed genes (DEGs) with false discovery rate adjusted *P* value < 0.05 when grown under 100% CO or 100% N_2_, respectively. Most of the DEGs were components of 23 gene clusters, suggesting switch between metabolisms via intensive expression changes in a relatively low number of gene clusters. Of the 9 significantly expressed gene clusters under 100% CO, CODH-II and ECH gene clusters were found. Only the ECH gene cluster was regulated by the CO-responsive transcriptional factor CooA, suggesting that others were separately regulated in the same transcriptional cascade as the ECH gene cluster. Of the 14 significantly expressed gene clusters under 100% N_2_, ferrous iron transport gene cluster involved in anaerobic respiration and prophage region were found. Considering that the expression of the temperate phage was strictly repressed under 100% CO, hydrogenogenic CO metabolism might be stable for *C. pertinax*.

**Electronic supplementary material:**

The online version of this article (10.1007/s00792-019-01091-x) contains supplementary material, which is available to authorized users.

## Introduction

Carbon monoxide (CO) is toxic for many organisms. However, some prokaryotes, called CO-utilizing microbes (carboxydotrophs), can use CO as the source of energy and carbon for their growth (CO metabolism) (Mörsdorf et al. 1992). Owing to the low redox potential (− 520 mV) of CO (Grahame and DeMoll 1995), anaerobic carboxydotrophs can couple CO oxidation to various reduction reactions such as sulfate to sulfide (sulfate-reducers), water to H_2_ (hydrogenogens), Fe^3+^ to Fe^2+^ (Fe[III]-reducers), and CO_2_ to acetate (acetogens) or methane (methanogens) (Oelgeschläger and Rother 2008; Sokolova et al. 2009). Many anaerobic and thermophilic carboxydotrophs have been isolated (Sokolova et al. 2009) from hydrothermal environments where CO is supplied by volcanic gas, photochemical and thermochemical decomposition of organic matter, and as a by-product of certain thermophiles (King and Weber 2007; Techtmann et al. 2009). Among them, 25 strains representing 13 genera (21 species) have been reported as hydrogenogenic carboxydotrophs (Sokolova and Lebedinsky 2013). Recently, in addition to these 25 strains, three thermophilic and hydrogenogenic carboxydotrophs, namely *Thermococcus barophilus* (Kozhevnikova et al. 2016), *Thermoanaerobacter kivui* (Weghoff and Müller 2016), and *Parageobacillus thermoglucosidasius* (Mohr et al. 2018), have been reported. Although a number of strains of *Moorella thermoacetica* have been isolated, only one strain (*M. thermoacetica* strain AMP) exhibits hydrogenogenic carboxydotrophy, whereas the other strains are acetogenic carboxydotrophs (Jiang et al. 2009). Because of their ability to use potentially toxic CO and produce H_2_ as the source of energy for other microbes, hydrogenogenic carboxydotrophs are assumed to be important ‘CO scavengers’ and primary producers in the environment (Sokolova and Lebedinsky 2013; Techtmann et al. 2009; Yoneda et al. 2013, 2015).

In anaerobic carboxydotrophs, CODH, with Ni in its active center, catalyzes the oxidoreductive interconversion between CO and CO_2_ (Ragsdale 2004). The function of CODH has been principally predicted by the genomic context of each CODH gene (*cooS*; Techtmann et al. 2012). A model of hydrogenogenic carboxydotrophs, *Carboxydothermus hydrogenoformans,* possesses five genes (*cooS*-*I* to -*V*) which code for the catalytic subunits of CODHs (CODH-I–V) on its genome (Wu et al. 2005). Based on the genomic context of the gene clusters including each *cooS* and/or empirical evidence, their functions are predicted as follows: CODH-I, energy conversion conjugated with ECH; CODH-II, NAD(P)H generation; CODH-III, carbon fixation in the Wood–Ljungdahl pathway conjugated with acetyl-CoA synthase (ACS); and CODH-IV, oxidative stress response (Wu et al. 2005; Svetlitchnyi et al. 2001). CODH-V does not conserve sequences responsible for its active center, and hence, its physiological function remains unknown (Inoue et al. 2013). *cooS*-I is arranged in a gene cluster with genes coding for an electron transfer protein (CooF) (Kerby et al. 1992), Ni insertion protein (CooC) (Kerby et al. 1997), a transcriptional factor (CooA) (Shelver et al. 1995), and ECH-related genes. In most hydrogenogenic carboxydotrophs, *cooS*-*I* and ECH-related genes form a gene cluster (CODH−ECH gene cluster) or are closely arranged (Sokolova et al. 2009). The hydrogenogenic CO utilization is performed by a complex of three enzymes; CODH-I, CooF, and ECH complex. CO is oxidized by CODH-I and the generated electron is transferred to CooF. Subsequently, this electron is coupled to proton reduction via the ECH complex, producing H_2_ and forming a proton gradient (Svetlitchnyi et al. 2001; Soboh et al. 2002).

Transcription of the genes in the CODH gene cluster in hydrogenogenic CO metabolism is activated by CO-responsive transcriptional factors CooA, RcoM (Kerby et al. 2008), and CorQR (Kim et al. 2015). Of these, CooA is well characterized in the hydrogenogenic carboxydotroph, *Rhodospirillum rubrum* (Aono et al. 1996; Roberts et al. 2004) and is present in most of the hydrogenogenic carboxydotrophs (Youn et al. 2004). CooA belongs to the cyclic AMP receptor protein family (Shelver et al. 1995). Homodimeric heme protein CooA is inactive in the absence of CO (Shelver et al. 1997). When CooA senses CO, ligand replacement in CooA leads to its conformational change making it active (Shelver et al. 1997). The active form of CooA can bind to the promoter region, thereby activating transcription through contact with RNA polymerase (Leduc et al. 2001). In the *R. rubrum* genome, CODH gene cluster and ECH gene cluster are closely arranged and the CooA-binding site is found upstream of both *cooF* in the CODH gene cluster and *cooM* in the ECH gene cluster (Fox et al. 1996; Rajeev et al. 2012). The previous study shows that CooA homologs are divided into two phylogenetically distinct groups (CooA-1 and CooA-2). CooA-1 is found in the majority of CooA possessing carboxydotrophs, whereas CooA-2 is found in some carboxydotrophs that possess multiple CODH gene clusters in their genomes (Techtmann et al. 2011). Furthermore, CO-binding assay of two CooA groups shows that both CooA-1 and CooA-2 are in their active forms in high CO concentration, whereas only CooA-2 is in active form even in low CO concentration (Techtmann et al. 2011). This difference in CO activation in the two CooA groups enables the bacterium to regulate multiple CODH gene clusters across wide range of CO concentrations (Techtmann et al. 2011). In addition to CooA, recent studies have reported other anaerobic CO-responsive transcriptional factors RcoM and CO-responsive regulatory system CorQR. Similar to CooA, RcoM, whose gene is adjacent to CODH and ECH gene clusters in the hydrogenogenic carboxydotroph *Rubrivivax gelatinosus* (phylum Proteobacteria) (Wawrousek et al. 2014), possesses a potential CO-sensor domain-containing heme (Kerby et al. 2008). CorQR pair is composed of CorQ with a DNA-binding domain of LysR-type transcriptional regulator (LTTR) family and CorR with 4-vinyl reductase domain instead of heme to sense CO (Kim et al. 2015). Genes encoding CorQR pair also flanked with CODH−ECH gene cluster in the hydrogenogenic carboxydotrophic archaeon, *Thermococcus onnurineus* (phylum Euryarchaeota) (Kim et al. 2015).

To date, only a few transcriptional studies about hydrogenogenic CO metabolism have been reported. A transcriptomic analysis of the sulfate-reducing carboxydotroph *Desulfovibrio vulgaris* using microarray reports that the expression of *cooS* is dependent on active CooA under low CO concentration (Rajeev et al. 2012). In addition, genome-wide primary transcriptomic analysis of *T. onnurineus* reports that the expression of *cooS* is significantly upregulated when they utilize CO as the source of energy for H_2_ production (Cho et al. 2017). However, there are limited data on comprehensive gene expression pattern of hydrogenogenic CO metabolism.

*Carboxydothermus pertinax* is isolated from an acidic hot spring in Japan (Yoneda et al. 2012). Despite of its hydrogenogenic carboxydotrophy (Yoneda et al. 2012), as per genome analysis, *C. pertinax* lacks genes encoding CODH-I catalytic subunit (CooS-I) and its transcriptional factor CooA-1 in their CODH-I−ECH gene cluster (Fukuyama et al. 2017). Furthermore, gene expression analysis in *C. pertinax* has shown that genes encoding CODH-II catalytic subunit (*cooS*-*II*) and distantly encoding the ECH catalytic large and small subunits are remarkably upregulated under 100% CO, suggesting that *C. pertinax* performs hydrogenogenic CO metabolism in which CODH-II couples with distal ECH (Fukuyama et al. 2018). Since *C. pertinax* possesses one CooA homolog (CooA-2) unlike *C. hydrogenoformans* possessing two CooA homologs, its transcriptional regulation from CO response is expected to be simpler than that of *C. hydrogenoformans*. Therefore, study of *C. pertinax* will further help us to understand the regulation mechanism of hydrogenogenic CO metabolism. In this study, we performed the whole transcriptome analysis of *C. pertinax* grown on pyruvate under 100% CO or100% N_2_ by RNA sequencing (RNA-Seq).

## Materials and methods

### Growth conditions for transcriptome analysis

To prepare RNA for transcriptome analysis, *C. pertinax* was grown at 65 °C in modified DSM medium 507 under a headspace of 100% CO or 100% N_2_ gas according to methods previously described (Fukuyama et al. 2018). We added sodium thiosulfate (final concentration, 1 g/L) as the terminal electron acceptor and sodium pyruvate (final concentration, 2 g/L) as the electron donor and carbon source to the medium in both the gas phase conditions to collect enough cells for analysis. Growth was assessed by direct enumeration of SYBR gold-stained cells collected on 0.2 μm black polycarbonate membrane filters (Advantec, Tokyo, Japan) using a fluorescent microscope (Olympus, Tokyo, Japan). The cells exponentially grown in pre-culture were inoculated to fresh medium and cultivated routinely.

### Transcriptome analysis of *C. pertinax*

When *C. pertinax* reached the late exponential phase as indicated by arrows (Fig. S1), 10 mL of the culture in both the conditions was collected and total RNA was extracted according to the methods previously described (Fukuyama et al. 2018). After removal of contaminating DNA using TURBO DNase (Invitrogen, Carlsbad, CA, USA), total RNA was purified using Agencourt RNAClean XP (Beckman Coulter, Brea, CA, USA) according to the manufacturer’s instructions. Quantification was performed in the Agilent 2100 Bioanalyzer with Agilent RNA6000 pico kit (Agilent Technologies, Santa Clara, CA, USA). Ribosomal RNA (rRNA) was depleted from the purified total RNA using Ribo-Zero^TM^ Magnetic Kit (Bacteria) (Epicentre, Madison, WI, USA) according to the manufacturer’s instructions. After rRNA removal, the purified total RNA, which was extracted from total 60 mL from the two replicates, was merged to obtain sufficient total RNA for RNA-Seq. Then, the merged total RNA was reverse transcribed to obtain double-strand cDNA (ds cDNA) using PrimeScript Double-Strand cDNA Synthesis Kit (TaKaRa Bio, Shiga, Japan) for RNA-Seq. The ds cDNA library was constructed with 75 bp paired-end libraries prepared by Nextera^®^ XT DNA sample prep kit (Illumina, San Diego, CA, USA) and sequenced using Illumina MiSeq system (Illumina). For reverse transcription quantitative PCR (RT-qPCR), the purified total RNA was reverse transcribed to single-strand cDNA (ss cDNA) using SuperScript III First-Strand Synthesis System (Invitrogen).

### RNA-Seq data analysis

Generated high-quality reads of *C. pertinax* with Q30 (sequence error rate lower than 0.1%) mapped to their draft genome (BDJK01000000) using Tophat version 2.0.13 (Trapnell et al. 2009) with Bowtie2 version 2.2.2 (mismatches $\le$ 2 bp) (Langmead and Salzberg 2012). We manually removed contaminated rRNA reads which showed high similarity to *C. pertinax* 5S, 16S, and 23S rRNA. Read count per gene under both conditions was estimated using featureCounts (Liao et al. 2014). Rarefaction curves for reads under both conditions were constructed using PAST ver.3.17 (Hammer et al. 2001). Gene expression levels were compared and normalized using the R statistical package edgeR (Robinson et al. 2010). DEGs were identified as significantly expressed under 100% CO or 100% N_2_ when their log2 fold change was > 1 or < − 1, respectively, and their FDR adjusted *P* value (*Q* value) was < 0.05.

### Analysis to predict the function of DEGs

As a unit of polycistronic transcription, a gene cluster was predicted based on the following criteria: (1) intergenic distance between genes was less than 300 bp, (2) genes were encoded in the same strand, and (3) the visual pattern of read alignment to a locus on *C. pertinax* genome. The Integrative Genomics Viewer (IGV) software was used to visualize the patterns of read alignment (Thorvaldsdottir et al. 2013). To predict the functions of DEGs, these protein sequences were annotated using BLASTp search (Altschul et al. 1990, 1997) with an e value of 1e^−5^ at an effective database size of 10^7^ against the COG database (Tatusov 2000). The upstream regions (300 bp) of the predicted gene cluster containing DEGs and solely transcribed DEGs were collected from the *C. pertinax* draft genome. The primary sigma factor recognition sequences were predicted using Bacterial Promoter Prediction Program (BPROM) with default parameters (Solovyev and Salamov 2011).

The promoter sequences were aligned separately for each motif (− 10 motif and − 35 motif). Logos were prepared using weblogo for visualization (Crooks et al. 2004). To discover motifs in the upstream regions, we used MEME ver5.0.2 program with default parameters (Bailey and Elkan 1994).

Prophage regions encoded in contigs (cut-off < 1500 bp) from the genomes of the three *Carboxydothermus* species except for the already analyzed *C. hydrogenoformans* (Wu et al. 2005) were predicted using PHAge Search Tool—Enhanced Release (PHASTER) (Arndt et al. 2016). To classify the predicted prophages in three *Carboxydothermus* species based on genome-wide similarities, a viral proteomic tree was generated by ViPTree (Nishimura et al. 2017b). The all-against-all distance matrix between viral reference genomes and the predicted phages was calculated on the basis of the normalized bit score of tBLAST*x* (*S*_*G*_) (Nishimura et al. 2017a), and the proteomic tree was built with BIONJ using the distance matrix in the ViPTree.

### RT-qPCR validation for the expression of CO metabolism-related genes

To validate the RNA-Seq expression data, 10 predicted CO metabolism-related genes were selected for RT-qPCR. RT-qPCR primers have been described in the previous study (Fukuyama et al. 2018). PCR amplification was performed according to the previous study (Fukuyama et al. 2018). We served same cDNA samples as RNA-Seq to RT-qPCR. The detection limit of the transcript levels of mRNA for target genes was 1.00 × 10^2^ copies/μL. Relative transcript amounts were calculated using *rrsD* (16S rRNA) transcripts as an internal standard. All RT-qPCR data represent the mean value of at least triplicate biological determinations.

### Accession number of the sequence

RNA-Seq data have been submitted to the DNA Data Bank of Japan Sequence Read Archive under the accession no. DRA007734.

## Results

### Overview of RNA-Seq

*Carboxydothermus pertinax* grew under both 100% CO and 100% N_2_ conditions (Fig. S1). Under 100% CO (carboxydotrophic growth), doubling time was 2.3 h with a final cell density of 8.25 × 10^7^ ± 1.05 × 10^7^ cells/mL. Under 100% N_2_ (heterotrophic growth), and doubling time was 1.9 h with a final cell density of 4.07 × 10^8^ ± 1.68 × 10^7^ cells/mL. Total RNA was extracted from cells at late exponential phase in both conditions and subjected to RNA-Seq (Fig. S1). A total of approximately 3.54 M and 3.74 M high-quality reads were obtained from 100% CO and 100% N_2_ conditions, respectively. The total number of the mapped reads to the *C. pertinax* draft genome was 2.28 M and 1.95 M with 100% CO and 100% N_2_, respectively (Table S1). Rarefaction curves of the mapped reads under both conditions showed that sequencing data from each condition was exhaustive to describe the transcriptional profile when read counts reached approximately 1.0 M (Fig. S2), suggesting that the count of mapped genes was sufficient for RNA-Seq analysis under both conditions.

In general, central component of bacterial transcription is multi-subunit-DNA-dependent RNA polymerase. When we searched for sigma factors in *C. pertinax*, 13 sigma factors (one primary sigma factor, 11 alternative sigma factors as sigma^70^ family, and one sigma^54^) were found (Table S2). Read count of the primary sigma factor was 6495 and 2855 under 100% CO and 100% N_2_, respectively. These read counts were an order of magnitude higher among the other sigma^70^ factors including sigma^70^ factors for sporulation under both conditions (Table S2). These results clearly indicated that the obtained RNA-Seq reads reflected expression patterns of exponentially growing cells.

**Fig. 1:**
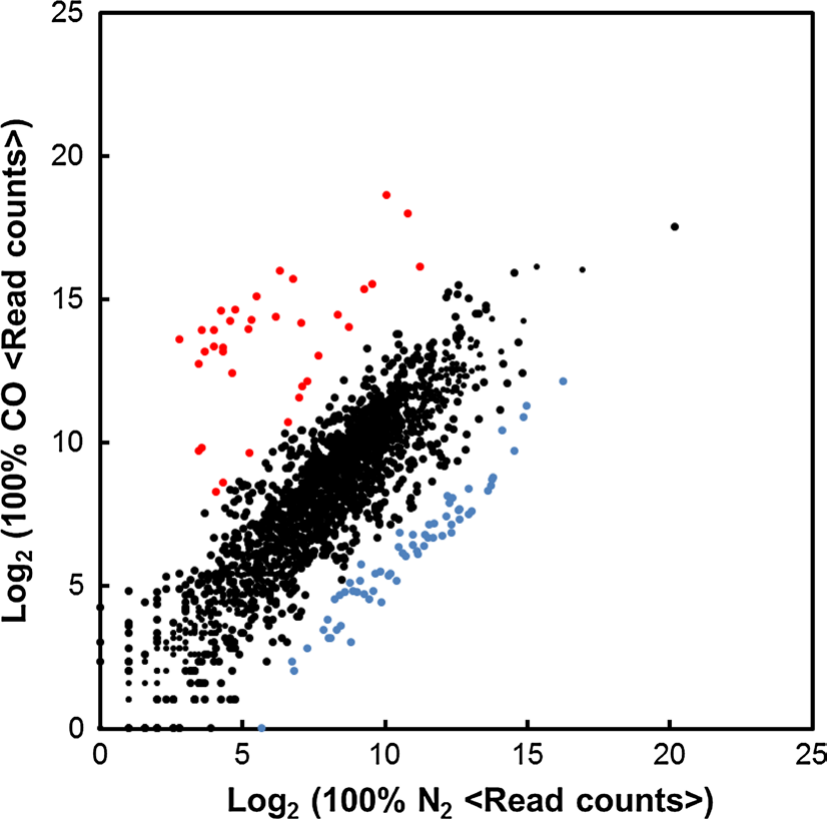
Comparison of read counts between *C. pertina*x grown under 100% CO and 100% N_2_. Each plot shows an open-reading frame. Red plots, upregulated DEGs; blue plot, downregulated DEGs; black, unchanged genes

Under 100% CO or 100% N_2_ conditions, 36 genes and 64 genes were identified as DEGs (*Q* value < 0.05), respectively (Fig. 1). A ratio of the 2577 genes in *C. pertinax* draft genome to the DEGs was no more than 1.4% under 100% CO and 2.5% under 100% N_2_ conditions, respectively. Seventy-four percent of the DEGs under 100% CO and 91% of the DEGs under 100% N_2_ were components of 9 and 14 gene clusters, respectively (Table 1). The remaining 8 DEGs under 100% CO and 6 DEGs under 100% N_2_ were solely transcribed. As significantly expressed gene clusters are expected to play key roles in both the conditions, we classified them into three categories based on average read counts per kilobase of the genes in the gene clusters containing DEGs (Table 1): very high (average read counts $\ge$ 10,000), high (average read counts $\ge$ 1000), and moderate (average read counts $\ge$ 100).

**Table 1:** Overview of gene clusters containing DEGs under 100% CO and 100% N_2_

Metabolism category	Locus tag	Predicted function	Fold change	No. of genes in gene cluster	No. of DEGs in gene cluster	Average read count		Expression category
						CO 100%	N_2_ 100%	
Significantly expressed gene cluster under CO 100%								
Energy conservation	cpu_03670-03750	ECH	480–1760	9	9	35,565	48	Very high
	cpu_14030-14050	CODH-II	70–380	3	3	97,573	405	Very high
	cpu_22890-22980	H_2_-uptake Ni–Fe-hydrogenase	20	1	10	471	118	Moderate
Carbohydrate metabolism	cpu_07810-07850	Anion/Na^+ ^ symporter, fumarate hydratase, and succinate dehydrogenase	460–960	5	5	12,770	23	Very high
	cpu_15750-15760	Succinate dehydrogenase	420–640	2	2	21,045	40	Very high
Transcriptional regulation	cpu_03570-03580	TetR/AcrR family transcriptional regulator	20–30	1	2	593	46	Moderate
	cpu_05930-05940	LysR-type transcriptional regulator	60–70	2	2	45,670	710	Very high
Unknown	cpu_03830-03840	Unknown	40	2	2	22,067	561	Very high
	cpu_23290-23300	Unknown	10	2	2	4326	58	High
Significantly expressed gene cluster under N_2_ 100%								
Energy conservation	cpu_18560-18580	Dimethyl sulfoxide reductase	30–40	3	3	205	6819	High
Prophage region	cpu_00830-00840	Myoviridae like prophage region	10–50	2	2	971	40,766	Very high
	cpu_00860-00940			9	9	465	15,716	Very high
	cpu_00950-01010			7	5	32	852	Moderate
	cpu_01060-01070			2	1	163	2382	High
	cpu_01080-01100			3	3	86	1504	High
	cpu_01110-01130			3	3	217	5618	High
	cpu_01140-01200			6	6	139	3903	High
	cpu_01220-01350			12	10	89	1428	High
Amino acid synthesis	cpu_11010-11030	Aromatic amino acid biosynthesis	20–40	3	3	74	2021	High
	cpu_11630-11700	Aromatic amino acid biosynthesis	20–30	8	4	26	292	Moderate
Membrane transport	cpu_18330-18350	Ferrous iron transport	10–20	3	2	1174	12,640	Very high
Unknown	cpu_13490-13500	Unknown	20–50	2	2	35	1372	High
	cpu_24930-24940	Unknown	20	2	2	1862	30,424	Very high

### Significantly expressed gene clusters under 100% CO or 100% N_2_

**Fig. 2:**
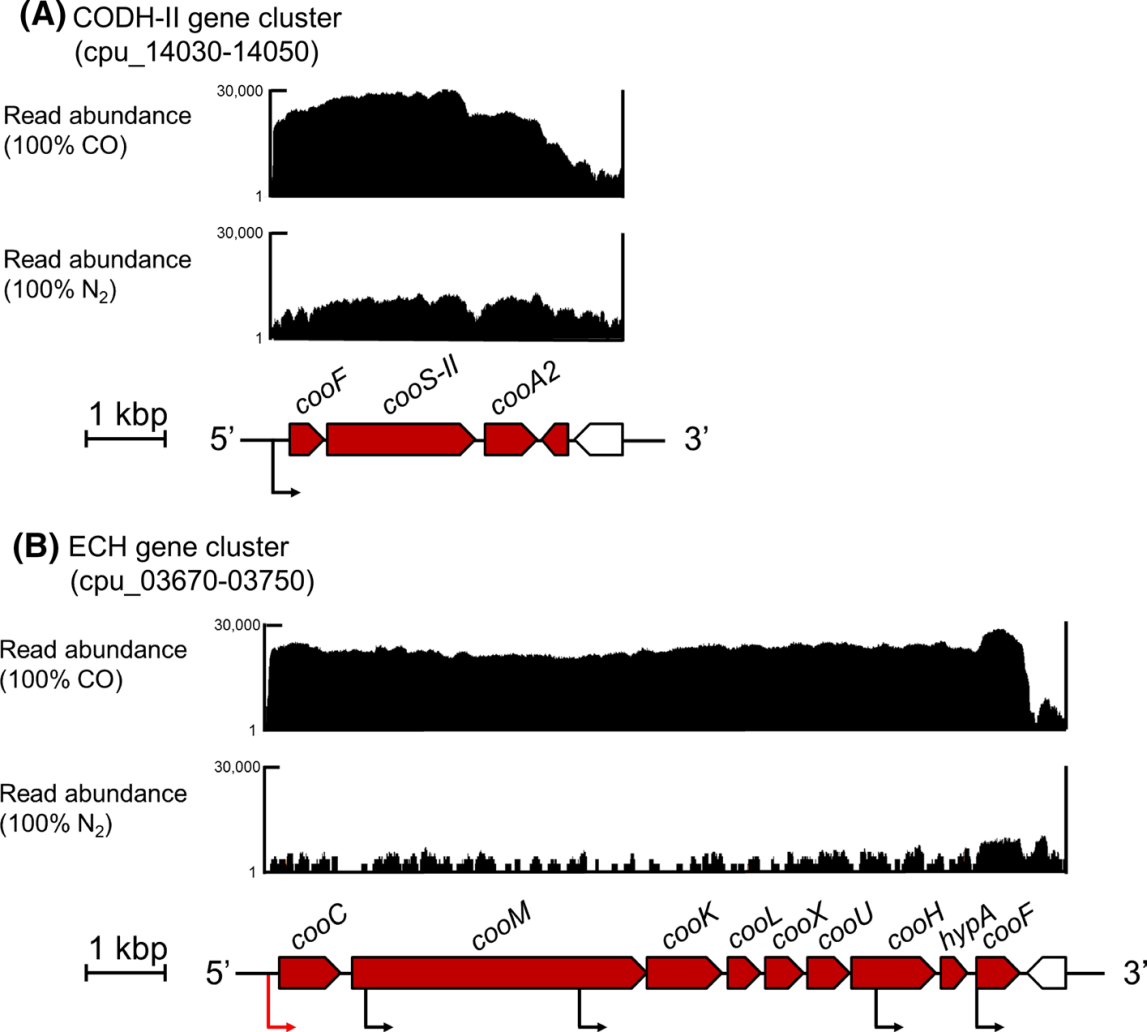
Transcriptomic read mapping pattern of the CODH-II gene cluster (**a**) and the ECH gene cluster (**b**). Part of genome-wide overview of reads mapped to the *C. pertinax* draft genome at samples in the CODH-II gene cluster (**a**) and the ECH gene cluster (**b**). Read abundance was displayed in a logarithmic scale of 1–30,000 by Integrative Genomics Viewer (Thorvaldsdottir et al. 2013). Red box, DEG under 100% CO; White box, not DEG under 100% CO. Positions of predicted sigma^70^ promoter sequence was predicted using BPROM with high linear discrimination function score (> 5; Solovyev and Salamov 2011). Black arrows, the position of predicted sigma^70^ promoter sequence. Red arrow, the position of predicted sigma^70^ promoter sequence with already known CooA-binding site

Of the 6 gene clusters in the ‘very high’ category under 100% CO (Table 1), the two gene clusters (cpu_14030-14050 and cpu_03670-03750; 70–380 and 480–1760 fold changes, respectively) were related to energy conservation and encoded CODH-II and ECH, which are essential proteins for hydrogenogenic CO metabolism (Fukuyama et al. 2018) (Fig. 2; Table S3). Furthermore, the two gene clusters were related to carbohydrate metabolism (460–960 and 420–640 fold changes, respectively; Table S3). Of these, the gene cluster (cpu_07810-07850) composed of five genes encoding divalent anion/Na^+^ symporter, fumarate hydratase α and β subunits, succinate dehydrogenase flavoprotein, and iron–sulfur subunit. The other gene cluster (cpu_15750-15760) composed of two genes encoding succinate dehydrogenase iron–sulfur and cytochrome b subunit. These gene products catalyze the oxidoreductive reaction between succinate and malate in tricarboxylic acid (TCA) cycle. The gene cluster (cpu_05930-05940; 60–70 fold changes) was composed of two genes encoding LTTR, involved in transcriptional regulation, and a hypothetical protein. In addition, a gene encoding another LTTR (cpu_07800) was also identified as a sole DEG (220 fold change). The function of the rest of the gene cluster (cpu_03830-03840) was not predicted from their gene products. Consequently, products of these six gene clusters were considered to play an important and specific role in their hydrogenogenic CO metabolism.

Next, we annotated three other categories (high or moderate; Table 1 and S3). These three gene clusters were composed of the following genes: two hypothetical genes (cpu_23290-23300; high), 10 H_2_-uptake Ni–Fe-hydrogenase genes (cpu_22890-22980; moderate), and TetR/AcrR family transcriptional regulator gene and sodium/phosphate symporter (cpu_03570-03580; moderate).

Of the four gene clusters in the ‘very high’ category under 100% N_2_ (Table 1 and S4), the two gene clusters (cpu_00830-00840 and cpu_00860-00940; 40 and 30–50 fold changes, respectively) were a part of the prophage region in *C. pertinax* (Table S4). From the viral proteomic tree generated using ViPTree, this prophage region (54 genes) was close to P2-like *Myoviridae* viruses, *Vibrio* virus X29 (NC_024369), and *Vibrio* phage phi 2 (KJ545483) (Fig. S4). The gene cluster (cpu_18330-18350; 10–20 fold change) was composed of two genes encoding ferrous iron transport proteins A and B (Lau et al. 2016). In addition, a gene (cpu_17460) encoding ferrous iron permease was identified as sole DEG (10 fold change). The function of the rest of the gene cluster (cpu_24930-24940) could not be predicted from their gene products. Products of these four gene products were also considered to play an important role in their heterotrophic growth.

Of the 10 gene clusters in high or moderate categories under 100% N_2_ (Table 1 and S4), the gene cluster (cpu_18560-18580; high) was involved in energy conservation and encoded dimethyl sulfoxide reductase catalytic molybdopterin subunit, iron–sulfur cluster protein, and integral membrane protein. The six gene clusters (cpu_01060-01070, cpu_01080-01100, cpu_01110-01130, cpu_01140-01200, and cpu_01220-01350; high, cpu_00950-01010; moderate) were found in the prophage region. Furthermore, the two gene clusters (cpu_11010-11030, high; cpu_11630-11700, moderate) were involved in aromatic amino acid biosynthesis. The function of the rest of the gene cluster (cpu_13490-13500; high) could not be predicted from their gene products.

### Expressions of genes involved in hydrogenogenic CO metabolism

Apart from the largely upregulated CODH-II gene cluster, RNA-Seq showed no significant expression changes of the CODH (CODH-III–V). In addition to ECH, *C. pertinax* possesses the H_2_-uptake Ni–Fe-hydrogenase gene cluster. In this gene cluster, read count of all genes were relatively low in both the conditions and only one gene (cpu_22950) encoding maturation factor for H_2_-uptake Ni–Fe-hydrogenase was identified as DEG.

To validate RNA-Seq, we performed gene expression analysis for 10 genes (*coos*-*IIA2F1* in CODH-II gene cluster, *cooS*-*III*–*V* in CODH-III–V gene cluster, *cooLH* in ECH gene cluster, and *hyaAB* in H_2_-uptake Ni–Fe-hydrogenase gene cluster) using RT-qPCR (Fig. S4). The relative transcript levels of five genes (*cooS*-*II*, *cooA2, cooF1, cooH,* and *cooL*) under 100% CO were 20–640-fold higher than those under 100% N_2_ (Fig. S4). Relative transcript levels of the three remaining genes (*cooS*-*III*–*V*) were not changed. Of them, relative transcript levels of *cooS*-*IV* were below the limit of detection (1.00 × 10^2^ copies/μL). Furthermore, relative transcript levels of the two genes (*hyaA* and *hyaB*) were relatively higher under 100% CO. Consequently, RT-qPCR analysis for these genes supported the RNA-Seq data (Fig. S4).

### Analysis of the regulation mechanism under hydrogenogenic CO metabolism

To predict the regulation mechanism under hydrogenogenic CO metabolism, we explored the transcription start site (TSS) from 300 bp upstream of 36 transcriptional regions (23 gene clusters containing DEGs and 13 sole DEGs), except for two transcriptional regions, which were significantly transcribed by the polar effect of their upstream. We assigned 36 TSSs from all promoter regions, regardless of their transcriptional categories (very high, high, and moderate). These 5′-untranslated region lengths of transcripts were ranged from 19 to 239 bp. The − 35 motif and − 10 motif (Wösten 1998) for primary sigma factor were strictly conserved among all the promoter regions in both the conditions (Fig. S5).

Among the 16 promoter regions of the significantly expressed transcriptional regions under 100% CO, the CooA-binding site was conserved only in the ECH gene cluster. Expect for the ECH gene cluster, we searched conserved binding motifs for transcriptional factor among other promoter regions. However, there is no consensus motif with a significantly low *E* value in the three transcriptional categories. Among the 20 promoter regions of the significantly expressed transcriptional regions under 100% N_2_, there is no consensus motif with a significantly low *E* value in three transcriptional categories.

## Discussion

In this study, we performed the whole transcriptome analysis of *C. pertinax* possessing only one CO-responsive transcriptional factor (CooA-2) as the first RNA-Seq report in hydrogenogenic carboxydotrophic bacterium. Total RNA from *C. pertinax* cells which grew under 100% CO and 100% N_2_ were compared to understand the regulation mechanism between hydrogenogenic CO metabolism and heterotrophic metabolism. RNA-Seq data suggested that *C. pertinax* switched its metabolism by considerable expression changes in a relatively low number of gene clusters.

Our previous study of *C. pertinax* shows that electrons from CO oxidation are mainly (62%) consumed to reduce H_2_O to H_2_; the remaining electrons are utilized in the reduction of thiosulfate by thiosulfate reductase (cpu_06910-06930) (Fukuyama et al. 2018). Genes involved in the transport of ferrous iron (very high category) were largely upregulated under 100% N_2_, suggesting that the ferrous irons were positively transported into cells in heterotrophic growth. Considering that ferrous irons were incorporated as hemes or iron–sulfur clusters in various energy-generating and regulatory proteins (Braun and Hantke 2011), *C. pertinax* could conserve energy via anaerobic respiration in the modified DSM medium 507 containing sodium thiosulfate. In contrast, in hydrogenogenic CO metabolism, transport of ferrous iron (very high category) was significantly and incompletely reduced to low expression (Table 1). Instead, both CODH-II gene cluster and ECH gene cluster were largely upregulated. In addition, expressions of the genes encoding thiosulfate reductase were relatively decreased (average read counts were decreased from 3018 to 1843). Therefore, owing to the sensitivity of heme protein to CO, *C. pertinax* would switch from anaerobic respiration to hydrogenogenic CO metabolism retaining a part of reducing power to the reduction of thiosulfate. The higher yields of *C. pertinax* grew under 100% N_2_ than 100% CO (Fig. S1) would be partly explained by a thermodynamic perspective about anaerobic respiration and hydrogenogenic CO metabolism. In addition, owing to the high sensitivity of CooA-2 to CO (Techtmann et al. 2011), *C. pertinax* would switch their metabolisms in response to low CO concentration.

In general, carboxydotrophs can fix CO_2_ or CO to acetyl-CoA via Wood–Ljungdahl pathway (Ragsdale 2004). In fact, transcriptome analysis of the acetogenic carboxydotroph, *Clostridium ljungdahlii,* shows that a gene cluster containing most of the genes for Wood–Ljungdahl pathway is significantly upregulated in their CO metabolism (Tan et al. 2013). In our RNA-Seq data, however, expressions of genes in CODH-III–ACS gene cluster were not upregulated regardless of CO addition. This result suggested that *C. pertinax* could fix carbon via other pathways in response to CO. Actually, a frameshift of CODH catalytic subunit gene in two hydrogenogenic carboxydotrophs suggested that carbon fixation via Wood–Ljungdahl pathway is not essential for hydrogenogenic CO metabolism (Wu et al. 2005; Omae et al. 2017).

In addition to Wood–Ljungdahl pathway, *C. pertinax* is predicted to possess an incomplete TCA cycle and an incomplete 3-hydroxypropionate cycle (Fukuyama et al. 2018). Among the incomplete TCA cycle-related genes, genes for fumarate hydratase (EC: 4.2.1.2; cpu_07820-07830 and cpu_21290-21300) and succinate dehydrogenase (EC: 1.3.5.1; cpu_078240-07850, cpu_15750-15760, and cpu_21260-21280) are multicopy (Fukuyama et al. 2017). Of these gene sets, one encoding fumarate hydratase (cpu_07820-07830) and succinate dehydrogenase (cpu_07840-07850 and cpu_15750-15760; Table S3) were largely expressed under 100% CO (very high category), implying that these gene clusters were upregulated in response to CO and products of these genes enhanced carbon fixation via a part of reductive TCA cycle. Furthermore, *C. pertinax* can conserve energy via their hydrogenogenic CO metabolism in which CO oxidation is coupled with H_2_ production and CO_2_ is generated as a by-product of this reaction (Yoneda et al. 2012; Fukuyama et al. 2018). Therefore, we hypothesized that, in addition to Wood–Ljungdahl pathway, *C. pertinax* fixed CO_2_ via reductive incomplete TCA cycle in response to CO.

In bacteria, self-cleaving activity of LexA repressor is stimulated by activated recombinase A (RecA) during response to DNA damage (SOS response) (Butala et al. 2009). As the temperate prophage also utilizes the bacterial SOS response system, host RecA promotes the entry of lytic phase when SOS response occurs in the host. In the prophage region, a gene encoding transcription repressor LexA (cpu_00820) was expressed under both conditions, whereas other genes such as DNA replication (cpu_00950) and many structure proteins were significantly downregulated under hydrogenogenic CO metabolism condition, suggesting that the lysis of the temperate phage was strictly repressed with 100% CO. In addition, these data might imply that hydrogenogenic CO metabolism is more stable than heterotrophic metabolism for *C. pertinax*.

Of the known anaerobic CO-responsive transcriptional factors, two transcriptional factors (CooA and RcoM for carboxydotrophic bacteria) possess heme domain (Kerby et al. 2008) and the other factor (CorQR for carboxydotrophic archaea) possesses 4-vinyl reductase domain to sense CO (Kim et al. 2015). Of the three transcriptional factors as DEGs under 100% CO, TetR/AcrR family transcriptional factor (cpu_03570) acts as the repressor when it senses cellular environmental dynamics (Deng et al. 2013). On the other hand, LTTRs (cpu_05930 and cpu_07800) is a widespread transcriptional factor in bacteria and acts as either activators or repressors of single or operonic genes (Maddocks and Oyston 2008). However, the LTTRs were not considered to be a CO–responsive transcriptional factor, because these possessed neither heme nor 4-vinyl reductase domains. When we explored transcriptional factor with known CO-sensing domain from *C. pertinax* using BLASTp search, only CooA-2 with heme domain was found. The CooA-binding site was conserved only upstream of the ECH gene cluster, strongly indicating that the ECH gene cluster was regulated by CO-responsive transcriptional factor CooA regulation. Collectively, it was suggested that only the ECH gene cluster was regulated by active CooA and others were regulated secondarily in the same transcriptional cascade as the ECH gene cluster. Otherwise, the expression of gene clusters, expect for the ECH gene cluster, was regulated by an undiscovered CO-responsive transcriptional factor.

## Conclusion

Our RNA-Seq analysis showed that *C. pertinax* switched its metabolism through intense expression changes of relatively low number of gene clusters. In heterotrophic metabolism, *C. pertinax* conserved energy via anaerobic respiration. On the other hand, owing to the inactivation of heme protein by CO, *C. pertinax* performed hydrogenogenic CO metabolism under 100% CO instead of anaerobic respiration retaining a part of reducing power from CO oxidation to the reduction of thiosulfate. Notably, genes in the temperate phage were strictly expressed under heterotrophic growth. This result might imply that hydrogenogenic CO metabolism might be more stable for *C. pertinax*. When *C. pertinax* switches heterotrophic metabolism to CO metabolism, of the significantly expressed gene clusters under 100% CO, only the ECH gene cluster was regulated by CooA regulation. In addition, no potential CO-responsive transcriptional factors were conserved in *C. pertinax*. These results suggested that only the ECH gene cluster was regulated by active CooA and others were separately regulated in the same transcriptional cascade as the ECH gene cluster.

## Electronic supplementary material

Below is the link to the electronic supplementary material.

Supplementary material 1 (DOCX 352 kb)

Supplementary material 2 (XLSX 22 kb)

## Abbreviations

CODH: Carbon monoxide dehydrogenase
ECH: Energy-converting hydrogenase
DEG: Differential expression gene
LTTR: LysR-type transcriptional regulator
