# Supplementary material for: Transcriptome analysis of a thermophilic and hydrogenogenic carboxydotroph Carboxydothermus pertinax
Source: Extremophiles. 2019 Apr 3;23(4):389–98. doi: 10.1007/s00792-019-01091-x (PMC6557876; doi:10.1007/s00792-019-01091-x)
Supplement: Supplementary file 1 — Supplementary material 1 (DOCX 352 kb) [file 792_2019_1091_MOESM1_ESM.docx]

**Supplementary materials for**

**Transcriptome analysis of a thermophilic and hydrogenogenic carboxydotroph *Carboxydothermus pertinax***

Yuto Fukuyama^1^, Kimiho Omae^1^, Takashi Yoshida^1^, Yoshihiko Sako^1^*

^1^ Division of Applied Biosciences, Graduate School of Agriculture, Kyoto University, Kyoto 606-8502, Japan

*To whom correspondence should be addressed. Tel: +81-75-753-6217; Fax: +81-75-753-6226; E-mail: [sako@kais.kyoto-u.ac.jp](mailto:sako@kais.kyoto-u.ac.jp)


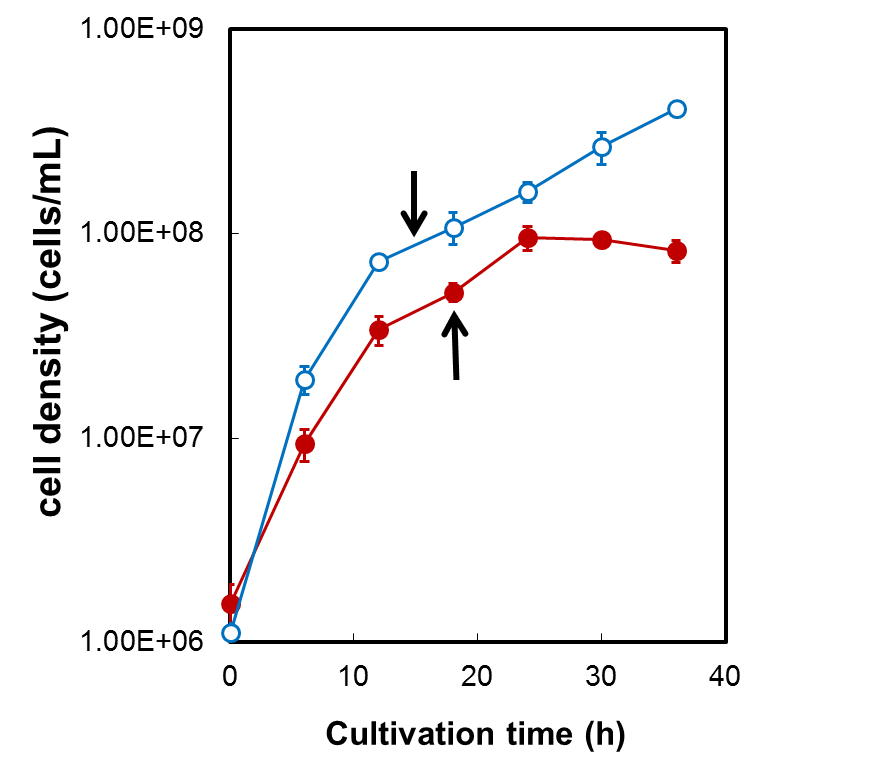


**Fig. S1 Growth of *C. pertinax* under 100% CO and 100%N_2_**

Red closed circles, cell density under 100% CO; blue open circles, cell density under 100% N_2_. Total RNA was extracted from *C. pertinax* when the cells reached late exponential phase as indicated by arrow. Plots represent the mean of three biological replicates. Error bars represent standard deviations.


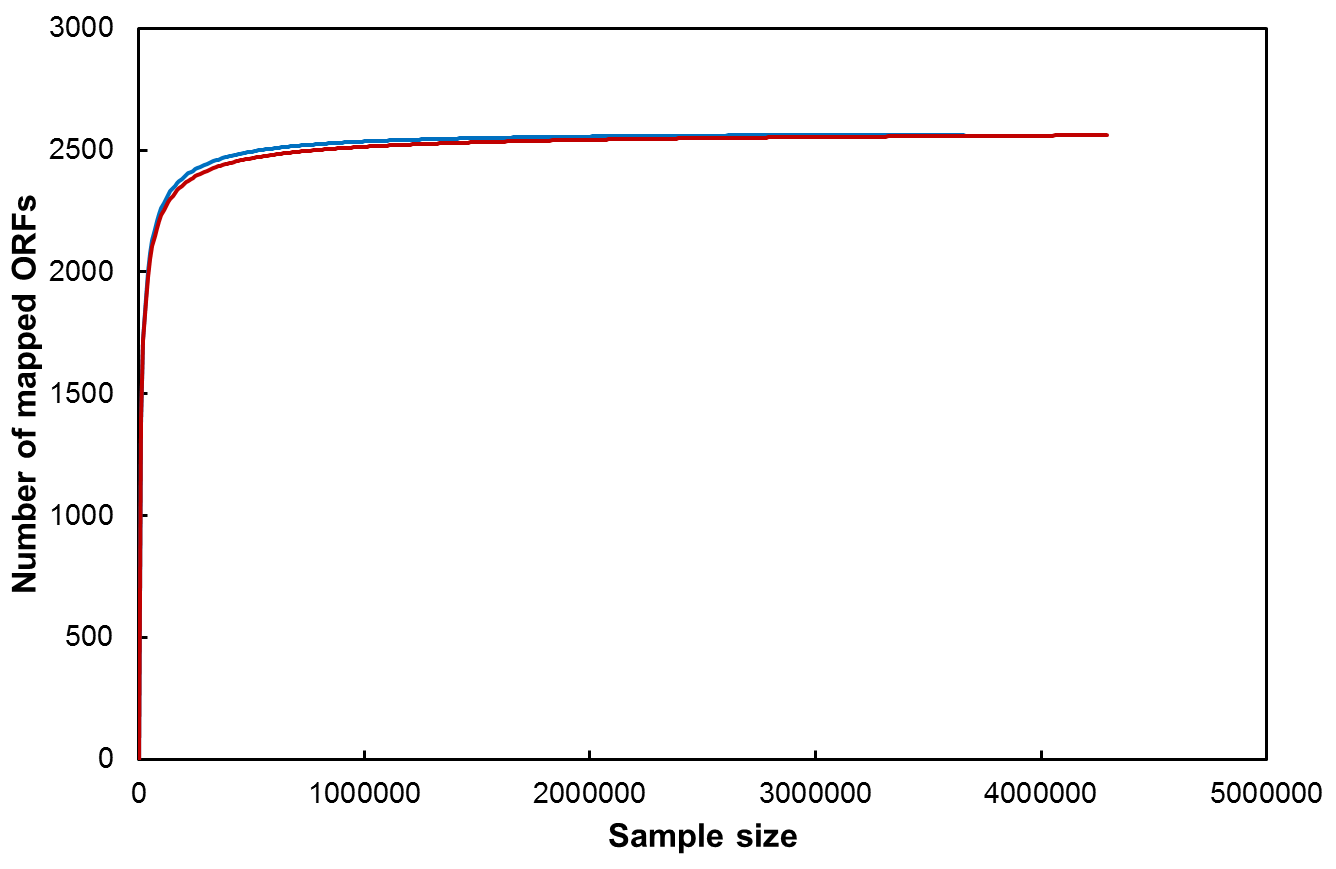


**Fig. S2 Rarefaction curves of mapped reads from the cells grown under 100% CO and 100%N_2_**

Red line, CO100%; blue line, N_2_100%.


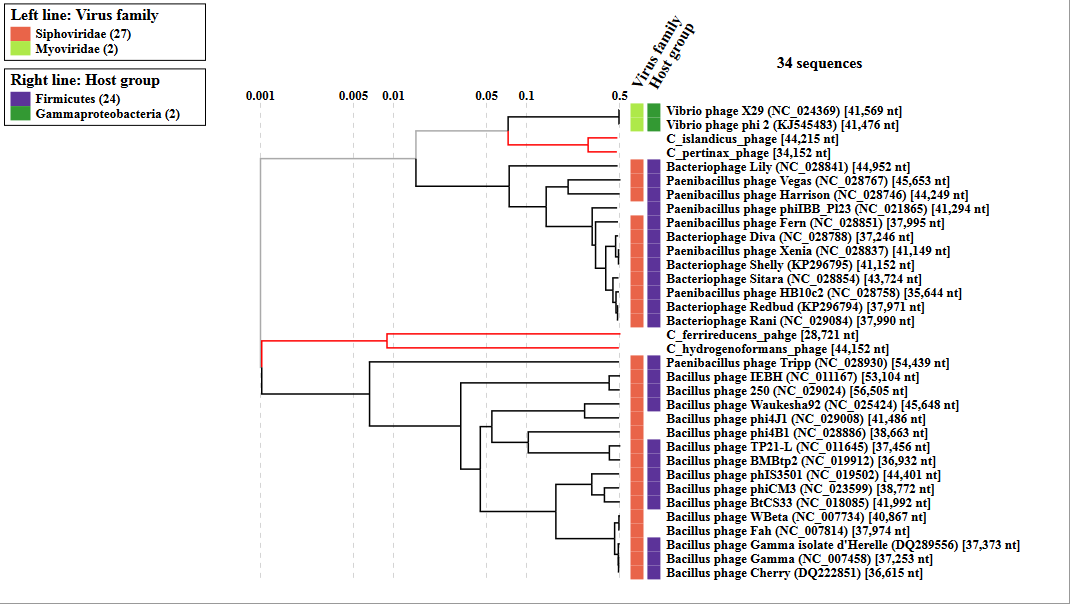


**Fig. S3 A part of proteomic tree with 4 phages from 4 *Carboxydothermus* species**

Branch lengths were logarithmically scaled from the root of the entire proteomic tree in VipTree database. Red branch represents phage in 4 *Carboxydothermus* species.


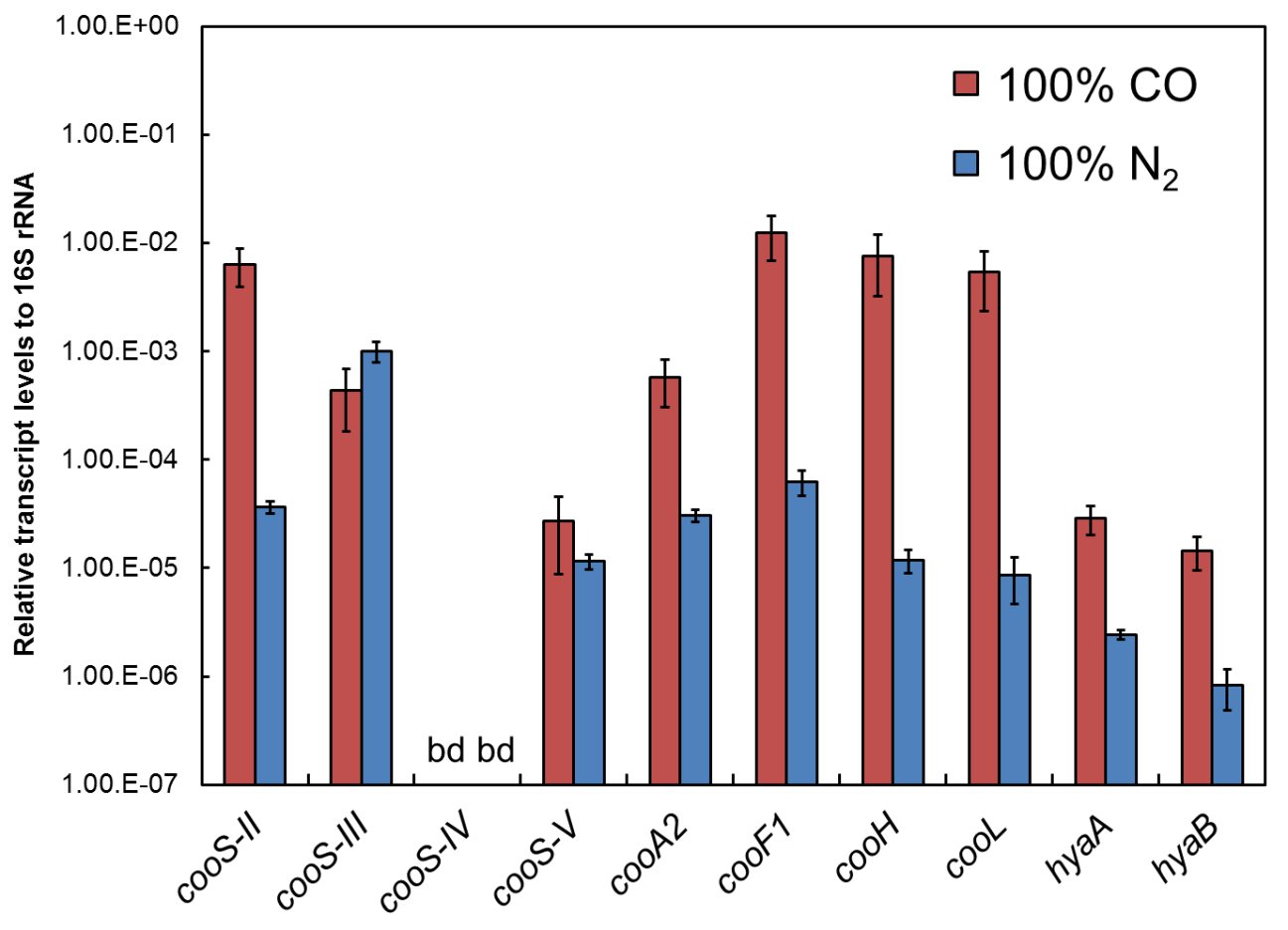


**Fig. S4 Transcript levels relative to 16S rRNA using RT-qPCR method under 100% CO and100% N_2_**

*C. pertinax* transcript levels relative to the 16S rRNA gene are shown. Red bar, cells grown under 100% CO; blue bar, cells grown under 100% N_2_. bd, below the limit of detection. Bars represent the mean of at least three biological replicates. Error bars represent standard deviations.


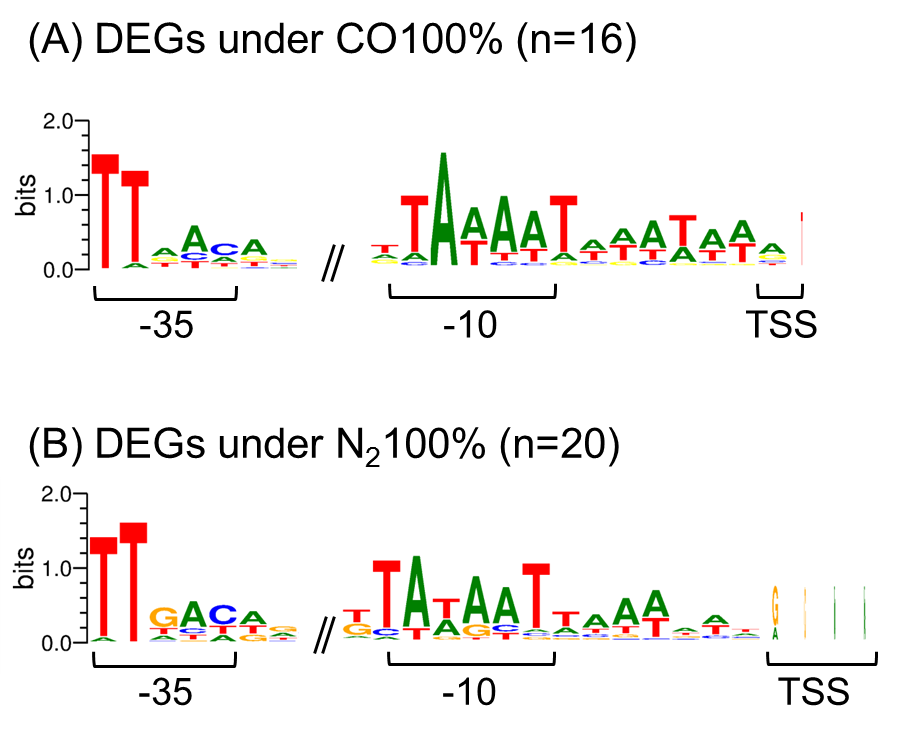


**Fig. S5 Consensus sequence for primary sigma factors among upstream regions of the significantlly expressed operons under 100% CO (A) and 100% N_2_ (B)**

The -35 and -10 motifs were identified from upstream regions of DEGs containing gene cluster and sole DEGs. The height of the letters is proportional to their frequency.
